# Supportive and demanding managerial circumstances and associations with excellent workability: a cross-sectional study of Swedish school principals

**DOI:** 10.1186/s40359-021-00608-4

**Published:** 2021-07-22

**Authors:** Roger Persson, Ulf Leo, Inger Arvidsson, Kerstin Nilsson, Kai Österberg, Carita Håkansson

**Affiliations:** 1 Department of Laboratory Medicine, Division of Occupational and Environmental Medicine, Lund University, 22185 Lund, Sweden; 2 Department of Psychology, Lund University, 22100 Lund, Sweden; 3 Centre for Medicine and Technology for Working Life and Society (Metalund), Lund, Sweden; 4 Centre for Principal Development, Umeå University, Umeå, Sweden; 5 Department of Public Health, Kristianstad University, Kristianstad, Sweden

**Keywords:** Education, Exhaustion, Leader, Self-rated health, Stress, Organisation, Wellbeing

## Abstract

**Background:**

The leadership of principals is important for school, teacher and student related outcomes. To be capable of doing their work (i.e., having sufficient workability), school principals need proper organisational preconditions, motivation, and good health. It is therefore concerning that some studies suggest that principals have a work situation that risks taxing their health and reducing their workability. However, few studies have examined the psychosocial working conditions of principals and no study has gauged principals’ workability. Accordingly, we decided to examine Swedish principals’ workability and their perceptions of eight demanding and five supportive managerial circumstances as well as the associations between managerial circumstances and reports of excellent workability.

**Methods:**

The participants comprised 2219 Swedish principals (78% women) who completed a cross-sectional web survey in 2018. A brief version of the Gothenburg Manager Stress Inventory (GMSI-Mini) gauged managerial circumstances. Workability was assessed with the workability score (0–10; WAS). Unadjusted and adjusted logistic regression analyses were used to examine associations between managerial circumstances and reports of excellent workability (WAS ≥ 9). Covariates were: length of work experience as a principal, school level, self-rated health, and general self-efficacy.

**Results:**

The results showed that circa 30% of the principals reported excellent workability. The GMSI-Mini results showed that role conflicts, resource deficits, and having to harbour co-workers’ frustrations were the most frequently encountered managerial demands. Meanwhile, cooperating co-workers, supportive manager colleagues, and a supportive private life were the most supportive managerial circumstances. Adjusted logistic regression analyses showed that role conflicts and role demands were associated with an increased likelihood of reporting less than excellent workability. In contrast, supportive managerial colleagues, a supportive private life and supportive organisational structures were associated with an increased likelihood of reporting excellent workability.

**Conclusion:**

Circa 30% of the participating principals perceived their workability to be excellent. Reducing role demands, clarifying the principals’ areas of responsibility and accountability in relation to other actors in the governing chain (role conflicts), striving for increased role clarity, and striving to find ways to separate work and private life, seem to be promising intervention areas if increasing principals’ workability is desired.

**Supplementary Information:**

The online version contains supplementary material available at 10.1186/s40359-021-00608-4.

## Background

The leadership of principals is important for school, teacher and student related outcomes [1, 2]. It is therefore noticeable that a number of international studies during the last four decades have identified numerous potential sources of stress and showed that being a principal often is taxing and potentially hazardous to one’s health e.g. [3–10]. Indeed, just as any employee, school principals need proper organisational preconditions, motivation, and good health, in order to retain their employment and to perform on a satisfactory level [11, 12]. However, the psychosocial work conditions of principals are still an understudied area of research [6, 7]. Existing psychosocial research on principals can collectively be described as being sprinkled over time, school levels, and school contexts, while at the same time being concentrated in the educational systems of English speaking countries. Thus, the temporal spacing between studies along with variations in local and/or national educational systems complicate both the extraction and application of knowledge obtained from previous studies. Accordingly, there is a need for more contemporary and comprehensive research studies that address school principals’ managerial circumstances and their associations to health and performance in other socio-cultural contexts (e.g., non-English speaking countries). From this perspective, it is conspicuous that the Swedish Work Environment Authority, which in 2009 and 2010 inspected the working conditions of principals in 52 municipalities in Western Sweden, observed problems with turnover, work overload, and performance, and that only approximately 10% of the visited school units paid sufficient attention to the principals’ working environment [13]. It is similarly conspicuous that we observed in a recent study that circa 25–30% of Swedish principals’ displayed signs of exhaustion [14], and that national data for the 2011/12, 2012/13 and 2014/15 semesters, showed that 29%, 22% and 27% of the principals changed school units, respectively [15]. Even if turnover rates may affect schools differently, and differ between age groups, school level, and the type of municipality (e.g., urban or rural) [15, 16], the high turnover rates and the high prevalence of exhaustion signs raise questions regarding the principals’ organisational preconditions. However, few efforts have been made to improve the principals’ work situation, and the knowledge gap in terms of how principals’ capacities to perform are affected by their work conditions warrants further investigation.

### Workability

Previous research on principals’ working conditions appear to have focused on assessing risk factors for perceived stress, low job satisfaction, or poor health [4, 6, 7, 17]. Few studies have attempted to identify salutary factors [18], that is, factors that presumably are beneficial for principals’ health, well-being, and performance. From this salutogenic perspective, however, workability appears to be a novel and potentially rewarding concept to apply for principals [19]. Introduced in Finland in the early 1980s, the workability concept was a response to a social need to adopt a more positive approach towards the problem of work disability that shortened individuals’ work careers and generated large costs for society [20]. Indeed, chronic health problems, including poor mental health, are associated with sick leave and reduced work performance [21, 22].

Essentially workability is a construct that gauges the individuals’ contemporary ability to work and is operationally defined by the Work Ability Index (WAI) [19, 23, 24]. The WAI contains seven items, some of which have several sub-items, and covers different aspects of work demands, employee health statuses and resources. While health and functional capacity are important features of workability, the opportunities and constraints of work itself, as well as knowledge, skills, competences, values, motivational factors, and life outside work, are all thought to contribute to an individuals’ workability [20].

While workability is considered a unidimensional construct, the authors of a German study proposed that WAI comprises two dimensions, reflecting in part subjectively estimated workability and in part objective health status [25]. In addition, a recent Iranian study found that their translation of WAI comprised three dimensions, that is, mental resources, self‐perceived work ability, and disease and health‐related limitations [26]. Nevertheless, several studies have independently suggested that item 1a in the WAI, also called the workability score (WAS) [27], and which assesses current workability in relation to the lifetime best, is a valid proxy for workability in large-scale studies [28–30].

Although the term workability may have different connotations in different settings (e.g., in occupational rehabilitation or in legal settings), in occupational research settings it is primarily viewed as a question of maintaining a balance between work demands and personal resources [20]. While often conceived as an outcome, or an intermediate variable, the theory underlying workability is similar to other models that emphasise the importance of a balance between demands and resources for health and well-being. Examples are stress-vulnerability models [31], the job demand-resource model (JD-R) of burnout [32], the cognitive activation theory of stress [33] and occupational balance [34].

Although managers occupy key positions within organisations, previous studies on managers’ workability are scant. However, in a Finnish study that entailed circa 1000 managers from diverse occupations, it was concluded that ageing managers and lower level managers were at risk of developing lower workability across 10 years [35], while the need to attend to psychosocial work conditions in organisations, in order to prevent poor workability among managers, was acknowledged [35]. To date, however, no study has gauged the workability of school principals or attempted to identify managerial circumstances that are favourable for the workability of principals.

### The work situation of principals in Sweden

In Sweden, being a principal is being part of a female-dominated profession. Depending on school level, the proportion of females varies between 93 (preschools) and 66% (compulsory school, upper secondary school and adult education) [36]. Also, since the 1990s, being a principal has meant being part of a decentralised and dual system of governance with national (e.g., parliament, ministry, and agencies) and local actors (e.g. municipalities and independent school owners) that sometimes have conflicting policies [37]. While actors on the national level govern by rules and regulations, municipalities, or other local school owners of independent schools, are responsible for organising education at all levels, from pre-schools to upper secondary schools, as well as municipal adult education and Swedish tuition for immigrants. According to the principals themselves, they work in a hierarchical education system, surrounded by layers of leadership both vertically and horizontally [37]. Vertically, upwards, they have to deal with superintendents and school owners. Vertically, downwards, they need to deal with assistant principals, teachers, and students. Horizontally, they need to interact with other principals from other school levels and school districts.

Swedish principals’ work roles are in part defined by several recent and nationwide educational reforms as well as general labour market decisions. For example, a new education act, new curricula for the compulsory and upper secondary schools, and a new grading system took effect in 2011, as did the professional certification of teachers in 2013. Also, the Swedish Working Environment Authority’s statute book on organisational and social work environment, which entered into force in 2016, added to the list of responsibilities [38]. Collectively, these reforms have substantially influenced how principals need to organise their work, including their responsibilities and accountabilities.

Another circumstance that defines the principals’ work role is the implementation of systems and procedures that build on New Public Management (NPM) [39] inspired ideas. These ideas have been promoted through an increased focus on accountability, decentralisation, marketisation, and management by objectives and results. Circumstances have forced Swedish principals to adopt a work role similar to private sector style managers [40]. In addition, trust-based management systems, which emphasise local governance and less control by the informal delegation of responsibilities and control to lower parties in the chain of command, have emerged as an alluring organisational logic [41, 42]. Effectively, this means that principals expect the leadership above them to be more trusting towards their own work, whereas they themselves need to be trusting when governing their subordinates. Trust is, however, a complicated matter and involves relationships, expectations and a willingness to take risks [43, 44]. Interestingly, both NPM and trust based management have in Sweden been introduced against the general backdrop of an industrial democracy tradition that emphasises participation in decision-making and employee influence [45, 46]. Arguably, the industrial democracy movement has contributed to making organisations in Sweden more flat and democratic but perhaps also more fuzzy and unclear. Furthermore, and at the level of local school districts or schools, the work of principals is characterised by responsibility and accountability in several areas, for example, pedagogical development, the work environment, staffing and budget, etc. [47, 48]. According to the most recent “Teaching and Learning International Survey (TALIS)”, which is coordinated by the Organisation for Economic Co-operation and Development (OECD), Swedish principals feel particularly stressed by too much administrative work, understaffing, and working with students with special needs [49, 50]. Studies have also identified the burden of living up to expectations that originates from various internal and external agents (e.g., superintendents, teachers, colleagues, other staff, parents and students) as a serious challenge [37]. Moreover, according to a governmental investigation, many problems are caused by role conflicts and demands within school districts (e.g., insufficient support and guidance from the municipal board or the owner of the schools) [51].

### Objectives

The purpose of the present study was to increase knowledge of Swedish principals’ working conditions by examining the extent to which demanding and supportive managerial circumstances were associated with perceived workability. Presumably, this knowledge will provide a needed basis for decisions on future actions aimed at improving principals’ workability. In light of the multitude of potential stressors a principal may be subjected to, and to manage this complexity, a brief version of the Gothenburg Manager Stress Inventory (GMSI) [52] was used to provide a quantitative summary of eight demanding, and five supportive circumstances that first and second line managers commonly face in their work (Table 1). The Workability score (WAS) was used as a single item measure of the current workability [27]. The following two research questions were asked:

- To what extent do principals report excellent workability as well as demanding and supportive managerial circumstances?
- To what extent are supportive and demanding managerial circumstances associated with reports of excellent workability?

**Table 1:** Description of the scales in the 32-item Gothenburg Manager Stress Inventory (GMSI)-Mini (n = 2219)

Domain and scale names	Description of item content (non-verbatim)	Number of items	Cronbach’s α
*Demanding managerial circumstances*			
Resource deficits	Insufficient possibilities to influence the allocation of resources to the organisation. Lacking resources due to the decisions of superiors, politicians or governmental authorities. Not enough resources to cope with peak loads	3	0.79
Organisational control deficits	Severe difficulties in implementing the decisions from higher levels in the organisation. Difficulties to follow how decisions are made in the organisation	2	0.73
Role conflicts	Conflicts between administrative work, organisational development and co-workers. Not enough time for organisational development. Difficulties in finding time to discuss the daily activities with colleagues	3	0.83
Role demands	Demanding responsibilities for (a) performance and quality; (b) personnel; (c) the work environment; and (d) organisational development	4	0.80
Group dynamics	Problems with feelings of safety and mutual trust within the co-worker group. Feelings of not knowing what is going on in the co-worker group. Co-workers having trouble accepting the common goals of work	3	0.79
Buffer-function	Demands of being a buffer between co-workers and higher levels in the organisation. Demands of having to explain bad or negative decisions that have been made by superiors. Superiors expect that you should be understanding and committed to accept decisions that are bad for you and your organisation	3	0.86
Co-workers	Demands on helping co-workers to organize and structure their work. Co-workers structures their work inadequately	2	0.83
Container-function	Demands of dealing with co-workers frustrations that work is psychologically challenging. Pressured co-workers burden you with their problems	2	0.87
*Supportiveng managerial circumstances*			
Supportive management	Trust that superiors, when needed, will help solving work environment problems for the co-workers. Experiencing that superiors express a genuine interest for the job and the problems I have as a leader	2	0.82
Cooperating co-workers	Feelings that co-workers want to take responsibility in their work. Feelings that co-workers have valuable knowledge that make the manager work easier	2	0.76
Supportive manager colleagues	Access to proper support from fellow school leader colleagues. Proper possibilities to reflect and discuss organisational issues with fellow school leaders colleagues	2	0.90
Supportive private life	The leisure time interests facilitates relaxation from work and associated problems. The leisure time really provides opportunities to rest and relax from work	2	0.87
Supportive organisational structures	Clearly defined authority in the work. Clearly defined area of responsibility and task as a leader	2	0.82

## Methods

### Study design

The present cross-sectional study collected data via an electronic survey and used a non-probability purposive sampling strategy that targeted Swedish principals at all school levels working at least 50%. Because there is no accessible official register of occupationally active school principals, participants were recruited via a list of e-mail addresses. This list had a nationwide reach and covered principals who during the period 2008–2017 had participated in training programmes funded and arranged by the Swedish National Agency for Education and run by different universities in Sweden. Sweden has a mandatory national principal training programme, stated by the Education Act 2010, for all newly appointed principals in the country. The programmes comprise 30 credits and requires three-years of studies on a level corresponding to 20% of the principals working time. The 30 credits are equally distributed across three courses: School legislation and the exercise of authority, Educational leadership, and Governance, organization and quality. After completion of the training programme, principals have the possibility to further their competence by participating in specialized leadership courses. The data was collected in the Swedish language and with the software Textalk Websurvey (www.textalk.se; Gothenburg, Sweden) between September 25 and October 23 of 2018. Up to four reminders were sent to non-responding participants. The participants were informed to carefully read the questions and that they should set aside at least 30 min to complete the survey, and that it was possible to save their responses if they were short on time.

### Participants

The participants in the present study are identical to the participants in our previous study that examined the prevalence rates of signs of exhaustion [14]. In brief, 9900 principals were invited via e-mail. Of these, 4640 potential responders either accepted (n = 2633) or declined (n = 2007) participation (i.e., a 47% response rate). Ultimately, 2317 responded, yielding a response rate of 23% in relation to all invited and 50% in relation to those who actively responded to the invitation. Of these, 2219 met the inclusion criteria: working at least half time (50%) while being employed in pre-schools (28%), pre- and compulsory school (5%), compulsory school (44%), upper secondary school (15%) or adult education (7%). Their mean age was 49.3 years (SD 7.4 years), 78% were women (n = 1724), 22% were men (n = 491), and 0.2% (n = 4) did not disclose their gender. Eighty-one percent had the job title principal (including the title pre-school director that since 2019 changed to pre-school principal) and 19% assistant principal. The majority of the participants (96.0%) reported working ≥ 90% of full-time. And 18.5% reported that during the preceding 12 months they had surpassed their scheduled work time on a daily basis, whereas 56% had done so a couple of days a week. Responders came from all 21 counties in Sweden and from 277 of the 290 municipalities. Nineteen percent had less than 3 years of work experience as a principal (women 21%; men 15%), 56% between 3 to 10 years of experience (women 57%; men 54%), and 24% more than 10 years of experience (women 21%; men 31%). Some 44.8% stated that they had not changed workplace during the last five years whereas 36.7% reported having changed workplace one time, 11.9% two times, and 6.6% three times or more. Municipalities (77%) and private share holding companies (11%) were the most common type of mandator. Other mandators (e.g., regional authorities, economic associations, foundations, cooperatives, etc.) employed the remaining principals (with no single mandator employing more than 3%).

### Outcome measure

The current workability, in comparison to the lifetime best, was assessed with item 1a from the Work Ability Index (WAI) [19, 23]. This single item is considered a valid proxy for workability in large-scale studies [28–30]. WAI item 1a is also called the Workability score (WAS) [27] and was responded to on an 11-step Likert-type scale with verbal anchors at the endpoints (0 = completely unable to work and 10 = work ability at its best). The item read: “*If you compare your current workability with your life time best, what score would you give your current workability; we assume that your workability at its best is valued as 10 points*”. Apart from using the score on the WAS as an outcome, for purposes of analysis, the score was also dichotomised into: 0 = “poor to good workability (0–8)”, and 1 = “Excellent workability (9–10)”. This cut-off score was selected as it previously was judged to correspond with good or excellent workability when assessed with the complete WAI inventory [53].

### Explanatory variables

The 32-item Gothenburg Manager Stress Inventory (GMSI)—Mini was used to gauge eight demanding and five supportive managerial circumstances, see Table 1 and Additional file 1 for item description and reliability estimates (Chronbach’s alpha). GMSI-Mini is a brief version of the GMSI, which is based on interviews with first and second line mangers and subsequent statistical analyses and testing of the interview-derived items [52]. GMSI-Mini was construed by selecting the most highly correlated items within each of 13 GMSI areas/dimensions (Österberg, K., personal communication). Accordingly, the 128 items in the GMSI were reduced to 32 items by selecting the items that in a previous validation of the full GMSI, across two different study samples, showed the highest mean correlation with the respective subscale in the original full GMSI [52]. The current version has previously been tested and applied in a pilot study on 251 principals in southern Sweden (Jansson and Wernbro, 2017; unpublished observations). In the present study, a confirmatory factor analysis (CFA), using a unit variance identification constraint (i.e., constraining the variance of each factor to 1), confirmed a good fit between the latent GMSI-Mini areas/dimensions and the observed data. The Comparative Fit Index (CFI) estimate was = 0.960 and the Root Mean Square Error of Approximation (RMSEA) estimate was = 0.040 (90% confidence interval = 0.038 to 0.042).

*Demanding managerial circumstances* were assessed in relation to the preceding six months and eight organisational areas: resource deficits, organisational control deficits, role conflicts, role demands, group dynamics, buffer functioning, co-workers, and container function. The items were responded to on a five-step scale: 1 = Never, almost never, 2 = Rarely, 3 = Sometimes, 4 = Often, 5 = Always, almost always.

*Supportive managerial circumstances* were assessed in relation to the preceding six months, four organisational areas, and one private life area: supportive management, co-operation with co-workers, supportive manager colleagues, supportive organisational structures and supportive private life. The items were responded to on a five-step scale indicating degree of agreement: 1 = Applies very poorly, 2 = Applies poorly, 3 = Applies to some extent, 4 = Applies well, and 5 = Applies very well.

For both demanding and supportive managerial circumstances, the mean score for each scale was used as the indicator. Higher mean scores indicated either experiencing more frequent demands or perceiving more support. The Cronbach’s alpha values ranged from 0.73 (Organisational control deficits) to 0.90 (Supportive manager colleagues), indicating an adequate internal consistency.

### Covariates

The covariates were selected conditional to their relevance for the workability construct, and the assumption that work performance is dependent on the interplay between situational circumstances (i.e., school level), motivational aspects (i.e., self-efficacy) and the capacity to perform (i.e., self-rated health and length of work experience) [11].

*Self-rated health* was assessed with one item that read, “*How do you assess your general state of health*” [54]. The response was rated on a five-point Likert scale: 1 = poor, 2 = fairly poor, 3 = neither good nor poor, 4 = fairly good, and 5 = very good. For purposes of analysis, this item was dichotomised: 0 = poor to neither good nor poor; 1 = fairly good to very good.

*Length of work experience as a principal* was compiled to a four step categorical scale (i.e., < 3 years, 3–5 years, > 5 years up to 10 years, > 10 years).

*School level* was compiled to a five step categorical scale (i.e., pre-school, pre- and compulsory school, compulsory school, upper secondary school, and adult education).

*General self-efficacy* was assessed with three items that have been used in previous research [55–57]. Yet, to comply with the current statement format of questioning the phrasing was slightly adjusted. Hence, preceded by a general question “To what extent do the following statements apply for you”, the three items read: “You can manage most unexpected events”, “You can solve most problems if you really want to”, and “Regardless of what happens in your life, you will feel that you probably will manage it”. All items had five response categories: 1 = never/hardly ever, 2 = seldom, 3 = sometimes, 4 = often, and 5 = always. The mean score (range 1–5) was used as a continuous predictor. Higher scores indicated greater self-efficacy. Cronbach’s alpha was 0.75.

### Statistical analysis

The IBM SPSS software (version 26.0.0.1) and the Jamovi software (version 1.2.27.0) were used for parallel statistical analysis. Statistical significance was set to two-tailed *P* values ≤ 0.05. An explanatory modelling approach was applied [58]. Spearman rank order correlations were used to estimate the strength of association between continuous variables. After confirming the linearity of the continuous variables with respect to the logit of the dependent variable [59], and that the correlations between variables were not too high, the probability for rating “poor to good workability” versus “excellent workability” was estimated with both unadjusted (model 0) and adjusted logistic regression analyses (models 1 to 4). Using the Jamovi software, and as a supplement to the visual inspection of the pattern of correlations between the predictor variables, the degree of multicollinearity among the predictors was estimated by calculating the variance inflation factor (VIF) for each predictor. In the final model, the VIF values ranged between 1.02 (i.e., self-efficacy, length of work experience as a principal, and school-level) and 1.35 (i.e., buffer-function), indicating no problems with multicollinearity. Results were expressed as prevalence odds ratios (OR) with accompanying 95% confidence intervals (CI). Nagelkerke R Square was used to illustrate how much variation in the outcome variable each adjusted model explained. Because the proportion of female and male principals was differentially distributed across school levels, we settled for including school level as a covariate (as opposed to making statistical adjustments for gender). However, sensitivity analyses were performed to explore potential differences between female and male principals as well as to estimate the effect of applying a lower cut-off score for the WAS score (i.e. score ≥ 8). Because age scores correlated weakly, or were unrelated, to the GMSI-Mini and workability scores, no statistical adjustments for age were made. Also, because the positive correlation between age and length of work experience was expected, and presumably part of the causal chain, we did not conduct age-stratified analyses.

## Results

**Fig. 1:**
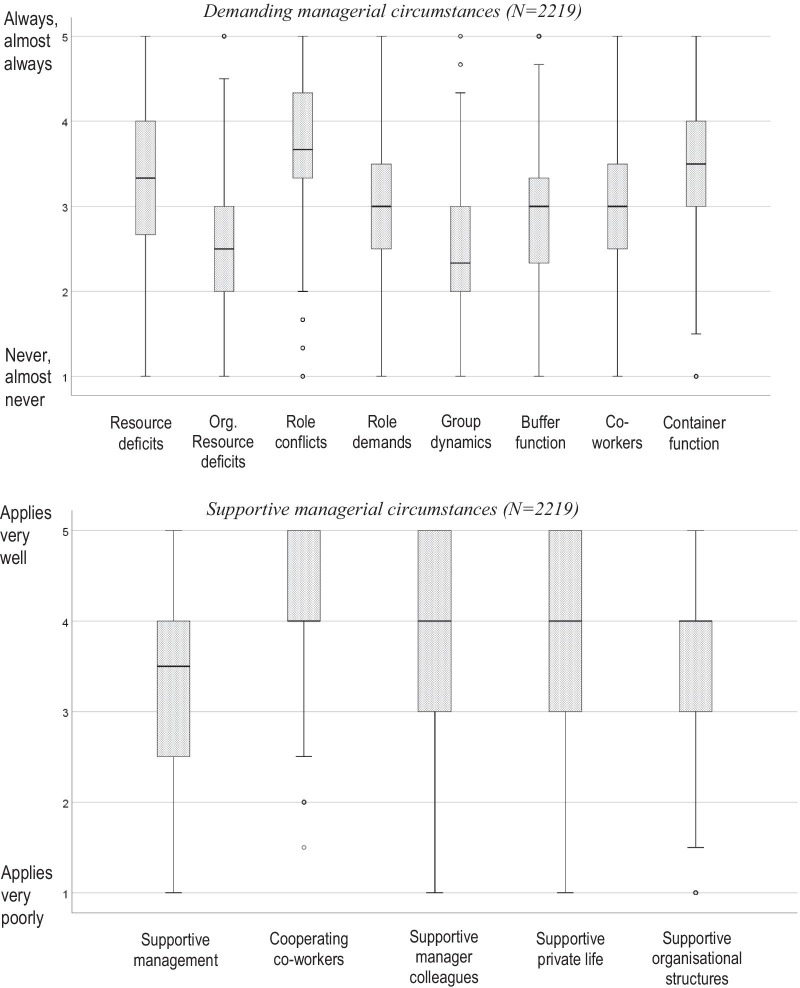
Descriptive box-and-whisker plot for the eight demanding and five supportive managerial circumstances in GMSI-mini. Rings represents observations beyond 1.5 times the inter quartile range (1.5IQR). The whiskers represents minimum and maximum values when the observations denoted by rings are removed. The demand items were responded to on a five-step scale indicating frequency: 1 = Never, almost never, 2 = Rarely, 3 = Sometimes, 4 = Often, 5 = Always, almost always. The support items were responded to on a five-step scale indicating degree of agreement: 1 = Applies very poorly, 2 = Applies poorly, 3 = Applies to some extent, 4 = Applies well, and 5 = Applies very well

Table 2 presents descriptive means, standard deviations, and inter-correlations between GMSI-Mini scores, WAS, self-rated health, general self-efficacy, and age scores. Figure 1 presents a descriptive boxplot of GMSI-Mini scores.

**Table 2:** Descriptive mean and standard deviation scores and spearman rank order correlations (n = 2119)

	M	SD	1	2	3	4	5	6	7	8	9	10	11	12	13	14	15	16	17
1. Age (years)	49.29	7.42	1																
2. Resource deficits^1^	3.41	0.97	.03	1															
3. Organisational control deficits^1^	2.53	0.89	.05*	.42**	1														
4. Role conflicts^1^	3.70	0.86	− .01	.39**	.40**	1													
5. Role demands^1^	3.07	0.80	.03	.35**	.36**	.56**	1												
6. Group dynamics^1^	2.37	0.75	− .01	.19**	.29**	.38**	.41**	1											
7. Buffer function^1^	2.91	0.93	.06**	.42**	.58**	.39**	.39**	.32**	1										
8. Co-workers^1^	2.91	0.74	− .09**	.14**	.16**	.26**	.26**	.41**	.21**	1									
9. Container function^1^	3.33	0.85	− .05*	.32**	.26**	.42**	.44**	.42**	.35**	.43**	1								
10. Supportive management^2^	3.22	1.09	− .05*	− .24**	− .41**	− .27**	− .26**	− .14**	− .42**	− .07**	− .17**	1							
11. Cooperation with coworkers^2^	4.20	0.66	.02	− .07**	− .12**	− .21**	− .24**	− .47**	− .15**	− .41**	− .27**	.10**	1						
12. Supportive mangers colleagues^2^	3.88	1.07	− .04	− .07**	− .16**	− .11**	− .15**	− .13**	− .19**	− .07**	− .09**	.36**	.18**	1					
13. Supportive private life^2^	3.83	1.02	.04	− .08**	− .11**	− .21**	− .24**	− .16**	− .10**	− .11**	− .18**	.13**	.19**	.18**	1				
14. Supportive org. structures^2^	3.65	0.92	.06**	− .18**	− .37**	− .27**	− .33**	− .23**	− .34**	− .14**	− .19*	.35**	.21**	.26**	.22**	1			
15. Workability score^3^	7.61	1.65	.01	− .17**	− .22**	− .32**	− .34**	− .26**	− .25**	− .13**	− .24**	.19**	.16**	.19**	.25**	.27**	1		
16. Self-rated health^4^	2.91	0.76	− .02	− .13**	− .11**	− .20**	− .21**	− .14**	− .14**	− .09**	− .17**	.12**	.13**	.15**	.37**	.18**	.33**	1	
17. Self-efficacy^5^	4.31	0.46	− .09**	− .03	− .10**	− .06**	− .18**	− .15**	− .09**	− .02	− .08**	.06**	.13**	.05*	.09**	.15**	.21**	.12**	1

*P < 0.05; **P < 0.001

M = Arithmetic mean value; SD = Standard deviation

^1^GMSI-Mini—demanding managerial circumstances: 1 = Never, almost never, 5 = Always, almost always

^2^GMSI-Mini—supportiveng managerial circumstances: 1 = Applies very poorly, 5 = Applies very well

^3^Workability: 0 = worst possible, 10 = best possible

^4^Self-rated health: 1 = poor health, 5 = very good health;

^5^Self-efficacy: 1 = low self-efficacy, 5 = high self-efficacy

The most frequently occurring demanding managerial circumstances were role conflicts, resource deficits, and having to fulfil a container function (e.g., having to manage co-worker frustrations). Oppositely, cooperating co-workers, supportive manager colleagues, and a supportive private life, were the most acknowledged supportive managerial circumstances.

The distribution of the principals’ WAS was: 0 = 0%, 1 = 0.1%, 2 = 0.5%, 3 = 1.6%, 4 = 2.3%, 5 = 5.5%, 6 = 10.2%, 7 = 22.8%, 8 = 26.7%, 9 = 17.7% and 10 = 12.3%. Accordingly, 30% reported a WAS score of 9 or 10 (i.e., excellent), whereas 60% rated their current workability as fair or good (scores 6–8) and only 10% gave ratings at or below the midpoint score of 5.

### Unadjusted logistic regression analyses

The unadjusted logistic regression analyses (model 0) showed that unadjusted odds following a 1-point increase in mean GMSI-Mini scores for the demanding factors were associated with a lower probability for reporting excellent workability (ORs between 0.43 and 0.75) (Table 3). Conversely, unadjusted odds following a 1-point increase in mean GMSI-Mini support scores increased the probability of reporting excellent workability between 32 and 74% (i.e., ORs between 1.32 and 1.74). In addition, the unadjusted odds for reporting excellent workability following a shift from poor to good self-rated health was OR = 3.22 (*P* < 0.001). And the unadjusted odds for reporting excellent workability following a 1-point increase in mean general self-efficacy score was OR = 2.50 (*P* < 0.001).

**Table 3:** Unadjusted logistic regression analyses between the continuous GMSI scores and the dichotomized workability score (WAS)

Independent variables	Total study sample (n = 2119)			Women (n = 1724)			Men (n = 491)		
	(OR increase with 95% CI, WAS score Poor to good vs. Excellent)			(OR increase with 95% CI, WAS score Poor to good vs. Excellent)			(OR increase with 95% CI, WAS score Poor to good vs. Excellent)		
	OR	95% CI	*P* value	OR	95% CI	*P* value	OR	95% CI	*P* value
*GMSI demanding circumstances, effect per unit increase in mean score (1–5)*									
Resource deficits	0.75	0.68–0.82	< 0.001	0.77	0.69–0.86	< 0.001	0.66	0.54–0.81	< 0.001
Organisational control	0.60	0.54–0.67	< 0.001	0.59	0.52–0.66	< 0.001	0.65	0.52–0.82	< 0.001
Role conflicts	0.50	0.44–0.55	< 0.001	0.49	0.43–0.56	< 0.001	0.50	0.40–0.63	< 0.001
Role demands	0.43	0.38–0.49	< 0.001	0.41	0.35–0.47	< 0.001	0.50	0.39–0.64	< 0.001
Group dynamics	0.54	0.47–0.61	< 0.001	0.55	0.47–0.63	< 0.001	0.51	0.28–0.67	< 0.001
Buffer-function	0.60	0.54–0.67	< 0.001	0.59	0.52–0.66	< 0.001	0.65	0.53–0.91	< 0.001
Co-workers	0.73	0.65–0.83	< 0.001	0.78	0.68–0.90	0.001	0.55	0.42–0.73	< 0.001
Container-function	0.58	0.52–0.65	< 0.001	0.60	0.53–0.68	< 0.001	0.51	0.40–0.64	0.001
*GMSI supportive circumstances, effect per unit increase in mean score (1–5)*									
Supportive management	1.39	1.27–1.51	< 0.001	1.43	1.30–1.57	< 0.001	1.22	1.01–1.48	0.037
Cooperating co-workers	1.59	1.37–1.84	< 0.001	1.57	1.33–1.85	< 0.001	1.69	1.22–2.36	0.002
Supportive colleagues	1.32	1.21–1.45	< 0.001	1.31	1.18–1.46	< 0.001	1.36	1.11–1.66	0.003
Supportive private life	1.61	1.45–1.78	< 0.001	1.60	1.43–1.79	< 0.001	1.62	1.29–2.03	< 0.001
Supportive org. structures	1.74	1.56–1.94	< 0.001	1.70	1.51–1.94	< 0.001	1.87	1.47–2.39	< 0.001
*Length of work experience as a principal*									
1–3 years of experience	1.02	0.77–1.34	.907	1.03	0.75–1.40	0.864	0.91	0.49–1.68	0.761
> 3–5 years of experience	0.91	0.69–1.18	.461	0.88	0.65–1.20	0.411	1.02	0.59–1.76	0.938
> 5 to 10 years of experience	0.94	0.74–1.20	.621	0.98	0.74–1.29	0.860	0.84	0.52–1.36	0.476
> 10 years of experience	Ref	–	–	Ref	–	–	Ref	–	–
*School-level*									
Pre-school	1.28	0.86–1.90	.223	1.33	0.82–2.13	0.246	0.71	0.20–2.50	0.599
Pre-and compulsory school	1.70	1.01–2.89	.047	1.61	0.87–2.99	0.127	2.87	0.75–6.94	0.145
Compulsory school	1.32	0.90–1.94	.151	1.39	0.87–2.22	0.137	1.12	0.58–2.18	0.734
Upper secondary school	1.30	0.85–2.00	.222	1.18	0.69–2.02	0.541	1.46	0.72–2.96	0.293
Adult education	Ref	–	–	Ref	–	–	Ref ara>	–	–
*Self-rated health*									
Poor, neither good nor poor(= 0) vs. good, very good (= 1)	3.22	2.44–4.24	< 0.001	3.21	2.35–4.37	< 0.001	3.24	1.71–6.03	< 0.001
*Self-efficacy, effect per unit increase in mean score (1–5)*									
Mean score	2.50	2.04–3.07	< 0.001	2.52	1.99–3.18	< 0.001	2.50	1.62–3.85	< 0.001

The unadjusted odds for school level and length of work experience as a principal did not substantially deviate from their reference levels (i.e., adult education and more than 10 years of work experience, respectively). The exception was that principals working in pre- and compulsory schools had an increased likelihood of reporting excellent workability as compared with principals working with adult education (OR = 1.70, p = 0.047).

### Adjusted logistic regression analyses

Adjusting for the GMSI-Mini scores and length of work experience as a principal (model 1), altered the estimates and the associated *P* values indicating an overlap between the independent variables (Table 4). Only five of the 13 GMSI-Mini variables retained statistical significance: role conflicts, role demands, supportive colleagues, supportive private life, and supportive organisational structures.

**Table 4:** Adjusted logistic regression analyses between the continuous GMSI scores and the dichotomized workability score (WAS; n = 2219)

Independent variables	Model 1 partially adjusted^1^			Model 2 partially adjusted^2^			Model 3 partially adjusted^3^			Model 4 fully adjusted^4^		
	(OR increase with 95% CI, WAS Poor to good vs. Excellent)			(OR increase with 95% CI, WAS Poor to good vs. Excellent)			(OR increase with 95% CI, WAS Poor to good vs. Excellent)			(OR increase with 95% CI, WAS Poor to good vs. Excellent)		
	OR	95% CI	*P* value	OR	95% CI	*P* value	OR	95% CI	*P* value	OR	95% CI	*P* value
*GMSI Demanding circumstances, effect per unit increase in mean score (1–5)*												
Resource deficits	1.11	0.98–1.25	.096	1.10	0.98–1.25	.115	1.12	0.99–1.26	.085	1.11	0.98–1.26	.108
Organisational control	0.90	0.78–1.05	.177	0.90	0.78–1.05	.187	0.89	0.76–1.03	.122	0.89	0.76–1.04	.132
Role conflicts	**0.76**	**0.65–0.88**	** < .001**	**0.76**	**0.65–0.88**	** < .001**	**0.77**	**0.66–0.90**	**.001**	**0.75**	**0.64–0.88**	** < 0.001**
Role demands	**0.70**	**0.59–0.83**	** < .001**	**0.69**	**0.58–0.82**	** < .001**	**0.69**	**0.58–0.82**	** < .001**	**0.73**	**0.62–0.87**	** < 0.001**
Group dynamics	0.86	0.72–1.02	.083	0.87	0.73–1.03	.107	0.86	0.72–1.03	.105	0.90	0.75–1.07	.222
Buffer-function	0.89	0.77–1.03	.119	0.91	0.78–1.05	.190	0.91	0.79–1.06	.226	0.92	0.80–1.07	.283
Co-workers	1.13	0.96–1.33	.156	1.13	0.96–1.33	.153	1.14	0.96–1.34	.137	1.09	0.92–1.29	.305
Container-function	0.87	0.75–1.01	.064	**0.85**	**0.73–0.99**	**.034**	0.86	0.74–1.00	.051	0.86	0.74–1.01	.057
*GMSI Supportive circumstances, effect per unit increase in mean score (1–5)*												
Supportive management	0.99	0.89–1.11	.904	1.00	0.89–1.11	.960	1.00	0.89–1.11	.924	1.00	0.90–1.12	.991
Cooperating co-workers	1.05	0.88–1.27	.576	1.04	0.86–1.25	.714	1.03	0.86–1.25	.732	1.01	0.84–1.22	.895
Supportive colleagues	**1.13**	**1.02–1.25**	**.018**	**1.13**	**1.02–1.26**	**.017**	**1.12**	**1.01–1.25**	**.030**	**1.13**	**1.01–1.25**	**.027**
Supportive private life	**1.32**	**1.19–1.47**	** < .001**	**1.32**	**1.19–1.48**	** < .001**	**1.23**	**1.10–1.38**	** < .001**	**1.23**	**1.10–1.37**	** < .001**
Supportive org. structures	**1.25**	**1.09–1.42**	**.001**	**1.26**	**1.10–1.43**	**.001**	**1.25**	**1.10–1.43**	**.001**	**1.22**	**1.06–1.39**	**.004**
*Length of work experience as a principal*												
1–3 years of experience	**1.47**	**1.08–2.00**	**.014**	**1.46**	**1.08–1.99**	**.015**	**1.43**	**1.05–1.94**	**.024**	**1.42**	**1.04–1.94**	**.028**
> 3–5 years of experience	1.18	0.88–1.58	.272	1.18	0.88–1.59	.263	1.16	0.87–1.56	.314	1.17	0.87–1.57	.311
> 5 to 10 years of experience	1.04	0.80–1.35	.793	1.04	0.80–1.36	.760	1.02	0.79–1.34	.861	0.99	0.76–1.30	.955
> 10 years of experience	Ref	–	–	Ref	–	–	Ref	–	–	Ref	–	–
*School-level*												
Pre-school	–	–	–	1.17	0.75–1.82	.492	1.18	0.75–1.83	.475	1.28	0.81–2.01	.287
Pre-and compulsory school	–	–	–	1.64	0.92–2.94	.096	1.71	0.95–3.08	.072	1.81	1.00–3.27	.052
Compulsory school	–	–	–	1.44	0.94–2.21	.092	1.45	0.95–2.22	.088	1.54	1.00–2.38	.051
Upper secondary school	–	–	–	1.24	0.77–1.97	.375	1.23	0.77–1.96	.397	1.29	0.80–2.08	.295
Adult education	–	–	–	Ref	–	–	Ref	–	–	Ref	–	–
*Self-rated health*												
Poor, Neither good nor poor (= 0) vs. good, very good (= 1)	–	–	–	–	–	–	**2.08**	**1.54–2.81**	** < 0.001**	**2.09**	**1.54–2.83**	** < 0.001**
*Self-efficacy, effect per unit increase in mean score (1–5)*												
Mean score	–	–	–	–	–	–	**–**	**–**	**–**	**2.01**	**1.60–2.53**	** < 0.001**

^1^ Model 1: Estimates are adjusted for GMSI-scores and length of work experience as a principal. Naglekerke R2 was 0.189.^2^ Model 2: Estimates are adjusted for model 1 and school level. Naglekerke R2 was 0.19.^3^ Model 3: Estimates are adjusted for model 2 and self-rated health. Naglekerke R2 was 0.21.^4^ Model 4: Estimates are adjusted for model 3 and self-efficacy. Naglekerke R2 was 0.23

Figures in bold are statistically significant *p* ≤ 0.05

Entering school level as a covariate only slightly altered the OR estimates (model 2). Additionally, when introducing self-rated health and subsequently self-efficacy, the general pattern of associations remained (model 3 and 4, respectively). Thus, five of the 13 GMSI-Mini variables retained statistical significance in the fully adjusted model. Accordingly, the odds for reporting excellent workability decreased with reports of increasing demands relating to role conflicts and role demands (Table 4). In contrast, the odds of reporting excellent workability increased with increasing reports of having supportive colleagues, a supportive private life, and supportive organisational structures (Table 4).

### Sensitivity analyses

Sensitivity analyses showed that male and female principals displayed a similar pattern of associations between managerial circumstances and workability (Table 3). Also, the lowering of the WAS cut-off score (i.e. ≥ 8) only slightly altered the pattern of OR estimates and associated *P* values, although demands relating to group dynamics reached statistical significance (Table 5).

**Table 5:** Sensitivity analysis: A lower cut off score for the workability score (WAS; (i.e., 0–7 vs. 8–10)

Independent variables	Total study sample (n = 2119)		
	(OR increase with 95% CI, WAS score 0–7 vs. 8–10)		
	OR	95% CI	*P* value
*GMSI Demanding circumstances, effect per unit increase in mean score (1–5)*			
Resource deficits	1.07	0.96–1.20	.258
Organisational control	0.97	0.84–1.12	.704
Role conflicts	**0.69**	**0.59–0.81**	** < .001**
Role demands	**0.80**	**0.68–0.94**	**.007**
Group dynamics	**0.82**	**0.70–0.96**	**.016**
Buffer-function	0.91	0.79–1.05	.198
Co-workers	1.13	0.97–1.33	.121
Container-function	0.87	0.76–1.01	.066
*GMSI Supportive circumstances, effect per unit increase in mean score (1–5)*			
Supportive management	0.98	0.88–1.08	.631
Cooperating co-workers	0.94	0.79–1.11	.451
Supportive colleagues	**1.19**	**1.09–1.31**	** < .001**
Supportive private life	**1.20**	**1.09–1.33**	** < .001**
Supportive org. structures	**1.28**	**1.13–1.44**	** < .001**
*Length of work experience as a principal*			
1–3 years of experience	1.31	0.98–1.76	.068
> 3 to 5 years of experience	1.12	0.85–1.48	.410
> 5 to 10 years of experience	1.18	0.92–1.51	.205
> 10 years of experience	Ref	–	–
*School-level*			
Pre-school	0.94	0.63–1.41	.762
Pre-and compulsory school	**1.77**	**1.01–3.09**	**.047**
Compulsory school	1.25	0.85–1.83	.256
Upper secondary school	1.19	0.78–1.82	.425
Adult education	Ref	–	–
*Self-rated health*			
Poor, neither good nor poor(= 0) vs. good, very good (= 1)	**2.02**	**1.60–2.56**	** < .001**
*Self-efficacy, effect per unit increase in mean score (1–5)*			
Mean score	**1.81**	**1.46–2.24**	** < 0.001**

Nagelkerke R square for the total model was 0.22

Figures in bold are statistically significant *p* ≤ 0.05

## Discussion

In the present study, we examined Swedish principals’ perceived workability and their perceptions of five supportive and eight demanding managerial circumstances. In addition, we examined the extent to which these managerial circumstances were associated with ratings of excellent workability, defined as scoring 9 or 10 on the workability score (WAS).

The results showed that circa 30% of the principals perceived their workability as excellent, which is markedly lower than the 45 to 56% observed among more than 2000 randomly selected employees and managers in a politically governed regional organisation in Sweden, who rated their workability on four consecutive times with a two year interval in between [53]. Likewise, the principals’ mean WAS (M = 7.6, SD = 1.7) was slightly lower than the mean levels as presented in a Swedish stratified random national sample (M = 8.25, 95% CI = 8.15 to 8.35) [28]. In contrast, when comparing the principals’ mean WAS score with occupationally active individuals with musculoskeletal complaints, drawn from various occupations [53], the WAS mean level and dispersion is identical (M = 7.6, SD = 1.7).

Furthermore, when compared with individuals on sick leave, the principals’ mean WAS scores are at least around 1-point higher. For example, in one Swedish study entailing individuals on long-term sick leave (> 90 days/year), the mean WAS score was circa 6.7 points [28]. In yet another Swedish study including women on long-term sick leave (> 60 days) and who worked in human service organisations, the mean WAS scores were 4, 5, and 5; at baseline, after 6 and 12 months, respectively [29]. Because the WAS correlates well with the total WAI score across age and gender groups [60], and is associated with subsequent sick leave in the Swedish population [28], the findings suggest that principals on average should have a slightly higher risk for developing subsequent sickness absence than individuals in the Swedish population. Unexpectedly, age scores exhibited almost zero correlation with WAS scores, a previously common finding across studies on workability [23, 35]. Likewise, the correlations between age scores and GMSI-Mini scores were at best weak.

The principals’ reported levels of role conflicts, role demands, resource deficits, and container function in GMSI-Mini, confirm that many principals perceive that they frequently encounter demanding managerial circumstances that originate from different administrative tasks and from different levels in the governing system. This observation aligns with the results from a recent interview study that explored the link between external expectations, health, and wellbeing among Swedish school principals [37]. Nevertheless, the principals seem content with their co-workers (i.e., cooperating co-workers), manager colleagues (i.e., supportive manger colleagues), and their possibilities for rest and relaxation outside work (i.e., supportive private life). However, the fact that many principals report that they have clearly defined areas of authority and responsibility (i.e., supportive organisational structures) seems to contradict their reports of frequently experiencing role conflicts (i.e., conflicts between administrative work, organisational development and co-workers, as well as having insufficient time to deal with organisational development and co-workers). However, as shown in the adjusted logistic regression analyses, these factors seem to make their own unique contributions to the reports of excellent workability. And it is of course possible that principals might find themselves in a situation in which they have formal authority and responsibility but few possibilities to influence the situation (e.g., governmental demands of documentation that sometimes are perceived to interfere with the principals’ possibilities to be pedagogical leaders)[37]. At any rate, our findings in GMSI-Mini seem to support the notion that the Swedish educational system encompasses conflicting organisational logics on both the national level and on the level of individual school districts and schools [51].

Interestingly, the relatively lower mean score on the GMSI-Mini supportive organisational management scale, signals that many principals appear to crumble in their trust of their superiors and are doubtful that their superiors have a genuine interest in their problems or would help them to solve their problems if needed. Thus, the recent calls for implementing more trust-based management systems [41, 42, 61] might be an appealing prospect for many Swedish principals. However, it is not clear whether trust-based management will solve the inherent organisational conflicts that exist in the Swedish system of educational governance. For example, the layers of leadership and the double governance seem to put principals in awkward positions when goals at the national level contradict goals at the local level [37]. It is similarly unclear to what extent the wish for trust based management is a move towards industrial democracy [45, 46] and a counterforce to the NPM [39] inspired ideas that currently underpin the educational system. These are ideas that emphasise accountability, decentralisation, marketisation, and management by objectives and results [40, 47, 48].

The adjusted logistic regression analyses showed that role demands and role conflicts were associated with reports of less than excellent workability, even after adjusting for the other GMSI-Mini scales, length of work experience as a principal, school level, self-rated health, and general self-efficacy. That the GMSI-Mini resource deficits score seem to be a less robust indicator for reporting excellent workability is perhaps a bit surprising. Speculatively, and since the definition of resource deficits implies that the principals have little direct influence over the situation, it is conceivable that they react to resource deficits with some resignation and thus do not become overly stressed when experiencing it—“it’s just the way things are”. In contrast, excellent workability was robustly associated with reports of having clearly defined areas of responsibilities and authority (i.e., supportive organisational structures), having good possibilities to unwind from work and work related problems (i.e., supportive private life), and having access to support from other manager colleagues (i.e., supportive colleagues).

In addition, that good self-rated health and high general self-efficacy were also robustly associated to excellent workability, and seem to have a larger impact on workability than managerial circumstances, agrees with a recent study from the Netherlands [62], underlining that health and motivational factors contribute to workability and that health is a fundamental resource in school leadership [37]. Peculiarly, having worked just one to three years as a principal was associated with a higher likelihood of rating excellent workability. Because short work experience as a principal implies being younger and recently employed, it seems plausible that their general fitness and/or enthusiasm of being new on the job may have coloured their workability ratings. In any event, the sensitivity analysis, which entailed lowering the cut-off for the WAS, confirmed the underlying pattern of associations between GMSI-Mini scores, self-rated health, and self-efficacy on the one hand, and on the other hand, the WAS.

### Methodological considerations

The use of a validated workability measure [28, 29, 60, 62] and the large sample size added to the internal validity of the study. And the external validity of the results is strengthened by the fact that the participating principals worked on different school levels, had varying degrees of work experience, and entailed responders from 277 municipalities (of 290) and all 21 counties in Sweden. However, a higher response rate would have been desirable. Only 4640 individuals (i.e., 47%) of the 9900 invited returned our invitation by responding either yes or no to participation in the study. Of these, 2633 accepted participation and 2007 declined participation. In the end, 2317 principals (i.e., 23% of all invited and 50% of those who actively responded to the invitation) completed the survey. The generally low response rate can in part be explained by over coverage. Because of high job turnover among Swedish principals [15], and because of the fact that the e-mail list we invited from was a compiled census covering the years 2008 to 2017, we have probably invited participants who no longer represented the target population at the time they were invited in 2018. Another contributing factor to the low response rate could be that the principals were too busy to take the time to complete our questionnaire. It should also be noted that our data reflects the situation before the covid-19 pandemic. However, according to the Swedish Schools Inspectorates yearly report for 2020, most schools, according to the principals themselves, have been able to follow the usual structure and scheduling of teaching even if the situation is strained [63]. Yet, it cannot be ruled out that overstressed principals with low workability have refrained from participating in our study. If this is the case, then the present results are likely to overestimate both the principals’ perceived level of workability and the prevalence rate of excellent workability. Nonetheless, and even if the number and definitions of principals vary across official statistics sources [36, 64], a comparison with national demographic data suggests that we have obtained a reasonably representative study sample as regards to the mean age and the general distribution of male and female principals across the various school levels [36, 65]. However, the cross-sectional study design does not allow conclusions on the directionality of effects. Since all data was collected via self-reports, common method bias in the form of interrater effects cannot be excluded and detract from the internal validity [66]. Finally, the large number of statistical comparisons warrants attention and makes it is advisable to focus on the overall pattern of relationships and to view single significance tests with caution.

## Conclusion

Circa 30% of the participating principals perceived their workability to be excellent. Role conflicts, resource deficits, and having to fulfil a container function were the most frequently encountered managerial demands. In contrast, cooperating co-workers, supportive manager colleagues, and a supportive private life were perceived as the most supportive managerial factors. Noticeably, even after adjusting for the principals’ self-rated health, self-efficacy, school level, and length of work experience as a principal, role conflicts and role demands were associated with ratings of less than excellent workability. In contrast, supportive colleagues, a supportive private life, and supportive organisational structures were associated with ratings of excellent workability. Thus, reducing role demands and clarifying the principals’ areas of responsibility and accountability in relation to other actors in the governing chain from the national to the local level, striving for increased role clarity, and striving to find ways to separate work and private life, all seem to be promising intervention areas if improving the principals’ working conditions and increasing their workability is desired. Due to the novelty of the here presented findings between managerial circumstances and workability among principals, and the cross-sectional study design, the potential practical significance of our observations remains to be evaluated.

## Supplementary Information

**Additional file 1**. Description of the items in GothenburgManager Stress Inventory-mini (GMSI-mini).

## Data Availability

The datasets generated and/or analysed during the current study are not publicly available since the ethical approval by the Regional Ethical Review Board specifies that crude data must not be published on the Internet. The data are available from the corresponding author on reasonable request.

## Abbreviations

CI: Confidence interval
GMSI: Gothenburg manager stress inventory
OR: Odds ratio
*P* value: Probability value
SD: Standard deviation
WAI: Workability index
WAS: Workability score
