# Supplementary material for: Supportive and demanding managerial circumstances and associations with excellent workability: a cross-sectional study of Swedish school principals
Source: BMC Psychol. 2021 Jul 22;9:109. doi: 10.1186/s40359-021-00608-4 (PMC8295455; doi:10.1186/s40359-021-00608-4)
Supplement: Supplementary file 1 — Additional file 1. Description of the items in GothenburgManager Stress Inventory-mini (GMSI-mini). [file 40359_2021_608_MOESM1_ESM.docx]

# **Description of the items in Gothenburg Manager Stress Inventory-mini (GMSI-mini)**

*Translated from Swedish by the researchers. GMSI-mini builds on items from GMSI (Eklöf, Pousette, Dellve, Skagert, & Ahlborg Jr, 2010).*

**Demanding circumstances** **in managerial work (22 Items)**

*(The items are responded to on a five-point Likert scale: 1 = Never, almost never, 2 = Rarely, 3 = Sometimes, 4 = Often, 5 =Always, almost always.)*

Below we describe some conditions that may occur in the work as a manager. If you think about the last six months: how often has the following occurred in your work as a manager?

1. That you have insufficient opportunities to influence how much resources your organisations get?

2. That your organisation has insufficient resources due to decisions from superiors, politicians or authorities?

3. That your organisation does not have the resources to manage peak loads?

4. That decisions made higher up in the organisation (by your municipality/individual mandators) are very difficult, or impossible, to implement in your organisation?

5. That you find it difficult to get an overview of the decision-making paths in the organisation?

6. That there is friction and opposition between administrative work, organisational development and contact with co-workers?

7. That you cannot spend enough time on organisational development?

8. That you have difficulty meeting your co-workers to discuss issues that arises in the daily work?

9. That the responsibility for results and quality is burdensome?

10. That personnel responsibilities are burdensome?

11. That the responsibility for the work environment is burdensome?

12. That the responsibility for organisational development is burdensome?

13. Problems with psychological safety and mutual trust within the employee group?

14. That you feel that you do not know what is happening in your staff group?

15. That co-workers have difficulty accepting the common goals that exist for work?

16. That you must be a buffer between higher levels of the organisation and your co-workers?

17. That you need to explain and justify "bad/negative" decisions that have been made by superiors?

18. That superiors expects you to be understanding and prepared to accept decisions that disadvantage you or your organisation?

19. That you must help co-workers plan and structure their work?

20. That co-workers have insufficient structure in their work?

21. That you get to receive co-workers' frustration that the work is mentally stressful?

22. That pressured co-workers burden you with their problems?

**Supportive (facilitating) conditions in managerial work (10 items)**

*(The items are responded to on a five-point Likert scale: 1 = Applies very poorly, 2 = Applies poorly, 3 = Applies to some extent, 4 = Applies well, and 5 = Applies very well.)*

Below we describe some conditions that can facilitate the work situation. Your task is to indicate to what extent you agree with that these conditions apply to you. Think about how it has been over the past six months:

1. I trust that my superiors, when needed, will help me solve work environment problems for my co-workers.

2. Supervisors show a genuine interest in what I do and the problems I face as a manager

3. I feel that my co-workers want to take responsibility in their work

4. I feel that co-workers have valuable knowledge that makes my work easier

5. When necessary, I can get good support from management colleagues

6. I have good opportunities to discuss with my colleagues and talk about the organisation.

7. My hobbies facilitate relaxation from work and its problems.

8. My leisure time really provides me the opportunity to rest and relax from work.

9. My powers in my managerial assignment are clear and distinct.

10. My area of responsibility and assignment as a manager is clear and delimited

**Reference**

Eklöf M, Pousette A, Dellve L, Skagert K, Ahlborg Jr G. (2010). Utveckling av ett variations- och förändringskänsligt frågeinstrument för mätning av stressorexponering, coping beteende och coping resurser bland 1:A och 2:A linjens chefer inom offentlig vård och omsorg. [The development of a variation- and change sensitive question instrument for measuring stressor exposure, coping behavior and coping resources among 1st and 2nd line managers in public health care]. *ISM-rapport 7*. Göteborg: Institutet för Stressmedicin; 2010. Retrived from: <https://www.vgregion.se/ov/ism/>
